# HS6ST1 regulates acute myeloid leukemia chemotherapy resistance via TGF-β1 signaling

**DOI:** 10.21203/rs.3.rs-8725671/v1

**Published:** 2026-02-13

**Authors:** Christina Termini, Kelsey Woodruff, Diya Patel, Jack Peplinski, Nicollette Setiawan, Matthew Hagen, Soheil Meshinchi

**Affiliations:** Fred Hutch Cancer Centre; Fred Hutch Cancer Centre; Fred Hutchinson Cancer Center; Fred Hutchinson Cancer Center; Fred Hutchinson Cancer Center; Fred Hutchinson Cancer Center; Fred Hutchinson Cancer Research Center

## Abstract

Despite therapeutic advances, relapse remains the leading cause of death in patients with acute myeloid leukemia (AML). Growth factor signaling controls AML survival, proliferation, relapse, and chemotherapy resistance. Here, we studied heparan sulfate proteoglycans, a class of molecules that bind growth factors via their heparan sulfate chains to change their signaling ability. Heparan sulfate-growth factor interactions are controlled by the addition of sulfate groups catalyzed by heparan sulfotransferases, such as those encoded by *HS2ST1* and *HS6ST1*. Using AML patient cohort analyses, we demonstrate that increased *HS6ST1* expression is associated with worse survival and increased relapse risk for AML patients harboring *KMT2A*-rearrangements. Using cell line derived xenografts, we show that AML cells depleted of *HS2ST1*, but not *HS6ST1*, have increased bone marrow leukemic burden. Further, AML cells depleted of *HS6ST1* are more sensitive to cytarabine than Control cells, suggesting that *HS6ST1* regulates AML chemotherapy resistance. Heparan sulfate antagonism with surfen synergized with cytarabine to further support AML cell death compared to cytarabine alone. Mechanistically, we demonstrate that *HS6ST1* depletion in AML cells reduces TGF-β1-mediated signaling, which diminishes cell survival upon cytarabine treatment. Together, our data show that *HS6ST1* promotes AML cell chemotherapy resistance by supporting TGF-β1 signaling.

## INTRODUCTION

Acute myeloid leukemia (AML) is the deadliest blood cancer, with a 5-year overall survival rate stagnating around 30% (1, 2). One aggressive form of AML occurs when patients harbor fusions of the lysine methyltransferase 2A (*KMT2A*) gene with various partners (3, 4). Daunorubicin or idarubicin and cytarabine (Ara-C) are frontline chemotherapeutic agents for AML (5, 6). While many patients achieve remission following initial chemotherapy treatments, disease relapse remains high because of the persistence of drug resistant cells that can expand following treatment (1, 7).

The AML growth factor milieu influences disease progression and AML chemotherapy resistance (8–10). Heparan sulfate proteoglycans are transmembrane proteins that facilitate growth factor signaling in normal and malignant cells (11–14). Heparan sulfate proteoglycans bear glycan chains that are composed of repeating disaccharide units (15). Heparan sulfates can be modified by the addition of negatively charged sulfate moieties at the *N-*, 2-*O*, 6-*O*, or 3-*O* positions. Sulfation modifications are catalyzed by enzymes encoded by the genes *NDST1–4, HS2ST1, HS6ST1–3*, or *HS3ST1–7*, respectively (12, 15). The type and amount of sulfate modifications present on the glycan chain influences heparan sulfate-growth factor interactions and signaling (16, 17). Heparan sulfate proteoglycans and heparan sulfate modifications have important roles in cancer cell adhesion, proliferation, migration, drug resistance, and vascularization (13, 18–21). Recent work identified syndecan-2 as an important regulator of hematopoietic stem cell quiescence via TGF-β1 signaling, and other work has demonstrated that heparan sulfate structure is important for B-cell maturation (22, 23). Several cytokines important for normal and malignant hematopoiesis, including TGF-β1, CXCL12, FGF1 and 2, and PDGF, bind heparan sulfate (24). However, the impact of precise heparan sulfation patterns in AML is largely undefined.

In this study, we show that heparan sulfation is dysregulated at the transcript and glycan levels in AML cells compared to normal hematopoietic cells. We identify an association between high *HS6ST1* expression and poor survival outcomes for *KMT2A-*rearranged AML patients. Using CRISPR-edited MOLM-13 cells, we demonstrate that *HS6ST1* is crucial for AML cell survival in response to Ara-C, and this occurs via TGF-β1 signaling. Our data highlights the critical function of heparan sulfation in AML, enabling us to expand current models of chemotherapy resistance by incorporating this crucial glycan modification.

## MATERIALS AND METHODS

### Study resources

Detailed information for resources used throughout this study is included in **Supplementary Table 1.**

### Patients and Samples

Samples were obtained from 2072 children and young adults (age 0–29 years) enrolled in clinical trials CCG-2961 (NCT00002798, n = 81), AAML03P1 (NCT00070174, n = 121), AAML0531 (NCT00372593, n = 795), and AAML1031 (NCT00372593, n = 1075) with written, informed consent collected from patients and their legal guardians in accordance with the Declaration of Helsinki. Each protocol was approved by the National Cancer Institute’s central institutional review board (IRB) and the local IRB for each participating institution. Clinical data were available for all 2072 patients, with 1874 of those patients also having accompanying survival and transcriptomic data, and analyses were performed for that cohort with complete data. 68 normal bone marrow (NBM) samples were used as controls for expression analysis.

### Expression Analysis

Batch-corrected mRNA data aligned to GRCh38 with STAR was used. Resulting normalized gene counts were converted into transcripts per million. Violin plots were generated with log10(TPM) using ggplot2 (v3.4.2). Wilcoxon test with Benjamini Hochberg adjustment was used to determine significance between AML subtypes and NBM expression levels for Heparan sulfation genes *HS2ST1* (ENSG00000153936), *HS6ST1* (ENSG00000136720), *HS3ST1* (ENSG00000002587), and *NDST1* (ENSG00000070614).

### Survival Analysis

Kaplan-Meier survival curves were generated using the survival (v3.5–5) and survminer (v0.4.9) packages in R (v. 4.3.2). Survival times were calculated from time of diagnosis. Competing events such as death or induction failure were removed from cumulative incidence calculations to determine relapse risk. Survival curves were generated by binning samples into above (high) and below (low) median expression of the gene of interest.

### Glycosaminoglycan profiling

Cryopreserved de-identified peripheral blood specimens from AML patients were obtained from the Fred Hutchinson Cancer Center/University of Washington Hematopoietic Diseases Repository. All participants provided written informed consent in accordance with the Declaration of Helsinki under the oversight of the Fred Hutch Institutional Review Office. Preparation and analysis was performed by the University of California San Diego GlycoAnalytics Core as previously reported (25).

### Mouse models

Animal procedures were performed in accordance with the Fred Hutchinson Cancer Center Institutional Animal Care & Use Committee (PROTO2100049). Mice were housed and maintained in the Fred Hutch Comparative Medicine facility; mixed-sex adult mice 8–12-weeks of age were used for all studies. Mice were bred in house or purchased from the Fred Hutch Translational Research Model Services core.

### Cell culture

MOLM-13, THP-1, and Kasumi-1 cells were obtained from the American Type Culture Collection and maintained per the manufacturer’s instructions. Cell lines were authenticated using the CLA IdentiFiler Plus PCR Amplification Kit.

### Cell line xenografts

Mice were irradiated using a Mark 1 cesium irradiator (225 cGy). The following day, mice were intravenously injected with 1×10^6^ MOLM-13 cells via tail vein. For leukemic burden studies, 14 days post-injection, mice were euthanized and bone marrow was isolated from one femur, lysed with ACK buffer, and processed in complete IMDM (IMDM + 10% FBS + 1% penicillin-streptomycin). For homing assays, 16 hours post-injection, bone marrow was isolated from two femurs and two tibias, lysed with ACK buffer, and processed in complete IMDM. Spleens were harvested, tissue was dissociated, lysed with ACK buffer, and processed in complete IMDM. Peripheral blood was collected into EDTA immediately prior to euthanasia, lysed using ACK buffer, and processed in 10% FBS/PBS. Lysed cells were stained using antibodies or isotype controls (**Supplemental Table 1**) and analyzed by flow cytometry (LSRFortessa X-50 or BD FACSymphony A5). Data were analyzed using FlowJo software (v10.10.0). Frequencies are displayed as percent of live cells. Investigators were not blinded. No randomization was used. Sample sizes were estimated using power analyses to quantify the number of replicates needed to achieve at least 80% power and detect a ratio of 1.2 vs. null hypothesis of 1.0 with significance level of α = 0.013.

### Histology

Organs were formalin fixed for 72 hours and washed with PBS. Femurs were decalcified for 14 days (0.5M EDTA, 4°C). Samples were paraffin-embedded and sectioned at 4 μm. Sections were stained with anti-human CD33 with nuclear counterstain. Broad regions of interest encompassing the full section (spleen and liver) or BM compartment, excluding the epiphysis (bone), were defined by a single observer. Primary classifiers were trained to exclude glass, fold and tear artifacts and (bone only) cortical bone. In the remaining tissue, the CD33^+^ area fraction was determined using the areaquant analysis plugin within HALO software (v3.6.4134).

### Lentiviral transductions

A 24-well plate was coated with retronectin (50 μg, 2 hours) and blocked for 30 minutes (2% BSA). 5×10^4^ MOLM-13 cells were seeded on the retronectin-coated wells and infected with lentiviral vectors containing guide RNAs targeting Control, *HS2ST1*, or *HS6ST1* (MOI = 25) (Supplementary Table 1). Cells were spin occulated (30 minutes, 1000 rpm, 32°C) and incubated for two days before the media was changed. 1–2 weeks after transduction, GFP^+^ cells were sorted using a BD FACSymphony S6 and expanded.

### *In vitro* chemotherapy treatment

2.5×10^5^ MOLM-13 cells were seeded in complete RPMI supplemented with DMSO vehicle or Ara-C to a final concentration of 0.5 μM and incubated at 37°C, 5% CO_2_ for 24 or 72 hours. Cells were then stained in 1X Annexin binding buffer with fluorochrome conjugated Annexin V and 7-AAD and analyzed via flow cytometry. For surfen experiments, cells were treated with surfen to a final concentration of 40 μM. For TGFβ-1 co-treatment experiments, cells were treated with recombinant human TGFβ-1 to a final concentration of 5 ng/mL.

#### RT-qPCR and RNA Sequencing:

RNA was isolated using the Qiagen RNeasy Micro Kit. For RT-qPCR analyses, RNA was reverse transcribed using the Applied Biosystems High-Capacity cDNA Reverse Transcription Kit. Gene expression was analyzed using an Applied Biosystems QuantStudio 5 PCR machine. For RNA sequencing, library preparation and sequencing was performed using a NextSeq 2000 P2–100. Gene set enrichment analysis was performed against the C5: Ontology Gene Sets. Data are deposited in GEO (GSE314673).

### Intracellular flow cytometry

Cells were stained with BD Fixable Viability Stain, washed, and fixed with BD Fixation/Permeabilization solution. Cells were stained with primary and secondary antibodies, washed, and analyzed via flow cytometry. Mean fluorescence intensity (MFI) was calculated using FlowJo.

### CellTrace Violet

2.5×10^5^ MOLM-13 cells were stained with CellTrace Violet according to manufacturer instructions, seeded in complete RPMI, and treated with either vehicle or human TGF-β1 to a final concentration of 5 ng/mL. Cells were incubated at 37°C, 5% CO_2_ for 72 hours, stained with 7-AAD and analyzed via flow cytometry.

### Western Blotting

1×10^7^ MOLM-13 cells were harvested and lysed in RIPA buffer supplemented with protease and phosphatase inhibitors. 10–25 μg of each sample was separated using gel electrophoresis and transferred onto a PVDF membrane. Membranes imaged using a Li-Cor Odyssey system after antibody staining. Quantification was performed using the Image Studio Lite software (v5.2.5).

## RESULTS

### Heparan sulfate is dysregulated at the transcriptional and glycan scales in AML

**Figure 1:**
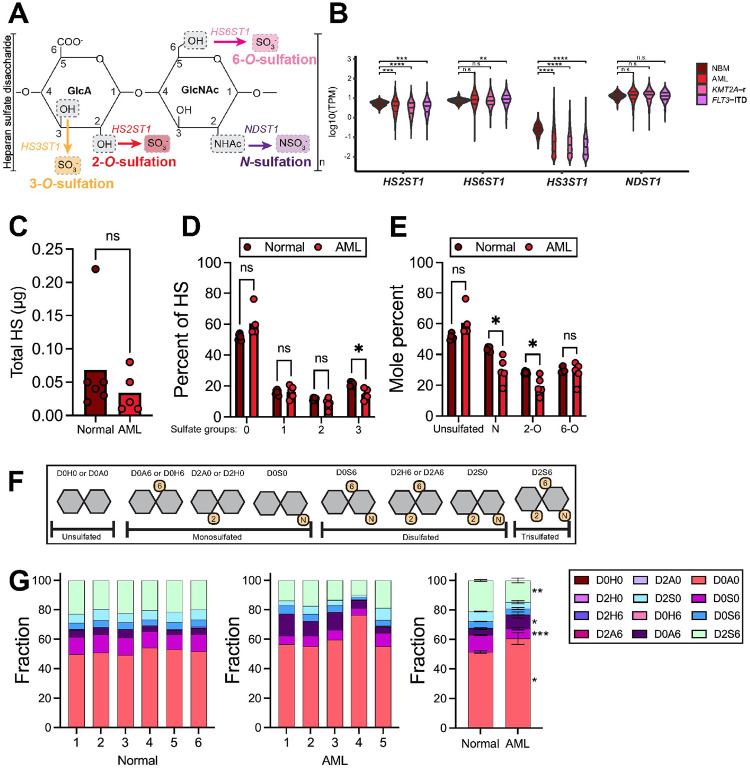
Heparan sulfate is dysregulated at the transcriptional and glycan level in AML patients. **(A)** Diagram depicting heparan sulfate chain modifications performed by *HS2ST1, HS3ST1, HS6ST1,* and *NDST1*. **(B)** Expression of *HS2ST1, HS6ST1, HS3ST1,* and *NDST1* in normal vs AML bone marrow analyzed from the TARGET AML database. (*n*=*68 NBM, n*=*2072 AML, n*=*466 KMT2A-r, n*=350 FLT3-ITD; statistics denote Wilcoxon tests followed by Benjamin Hochberg adjustments; *p<0.05, **p<0.01, ****p<0.0001). **(C)** Total heparan sulfate, **(D)** the fraction of HS bearing 0, 1, 2, or 3 sulfate modifications, and **(E)** the fraction of unsulfated, *N*-, 2-*O*, and 6-*O* sulfated HS was quantified using liquid chromatography mass-spectrometry in normal and AML PBMCs. (*n*=*6 normal PBMC, n*=5 AML PBMC; Statistics denote an unpaired t-test or multiple unpaired t-tests followed by a Holm-Šídák correction; *p<0.05). **(F)** Schematic depicting possible sulfation combinations on heparan sulfate disaccharides. **(G)** Specific heparan sulfate disaccharide composition in PBMCs isolated from normal or AML individuals, and, at right averaged disaccharide composition among analyzed specimens. (*n*=*6 normal PBMC, n*=5 AML PBMC; error bars show SEM; statistics denote a MANOVA; *p<0.05, **p<0.01, ***p<0.001).

We first assessed the expression of heparan sulfotransferase genes *HS2ST1*, *HS3ST1*, *HS6ST1*, and *NDST1* in healthy individuals and AML patients from the Therapeutically Applicable Research to Generate Effective Treatments (TARGET) database (Fig. 1A). Bulk RNA sequencing revealed significantly lower *HS2ST1* and *HS3ST1* expression in AML patient bone marrow mononuclear cells than normal bone marrow (NBM) mononuclear cells, while *HS6ST1* and *NDST1* expression were similar among these groups (Fig. 1B). AML patients with *KMT2A-*rearrangements and *FLT3-*ITD mutations expressed significantly less *HS2ST1* and *HS3ST1* than NBM cells. In contrast, patients harboring *FLT3-*ITD mutations expressed significantly more *HS6ST1* compared to NBM, while *NDST1* expression was similar between these groups (Fig. 1B). These data suggest that the transcriptional profile of heparan sulfotransferase genes differs in AML and NBM cells.

We next used liquid chromatography mass spectrometry to analyze heparan sulfate modifications of peripheral blood mononuclear cells (PBMCs) from normal patients and AML patients. AML patient characteristics are detailed in **Supplemental Table 2**. Total heparan sulfate amounts were similar in AML and normal cells (Fig. 1C). Each heparan sulfate disaccharide can bear zero, one, two, or three sulfate groups. Heparan sulfate disaccharides containing three sulfate groups were less frequent in AML PBMCs compared to normal PBMCs (Fig. 1D). AML PBMCs had lower fractions of *N-* and 2-*O* heparan sulfate compared to normal PBMCs (Fig. 1E). AML PBMCs had significantly less D0S0 *N-*monosulfated and D2S6 trisulfated disaccharides and significantly more D0A0 unsulfated and D0A6 6-*O* monosulfated disaccharides compared to normal PBMCs (Fig. 1F–G). Taken together, these data suggest that AML cells express distinct heparan sulfate landscapes with fewer sulfate modifications compared to normal cells.

#### Increased HS6ST1 expression is associated with worse survival outcomes in KMT2A-rearranged AML patients

**Figure 2:**
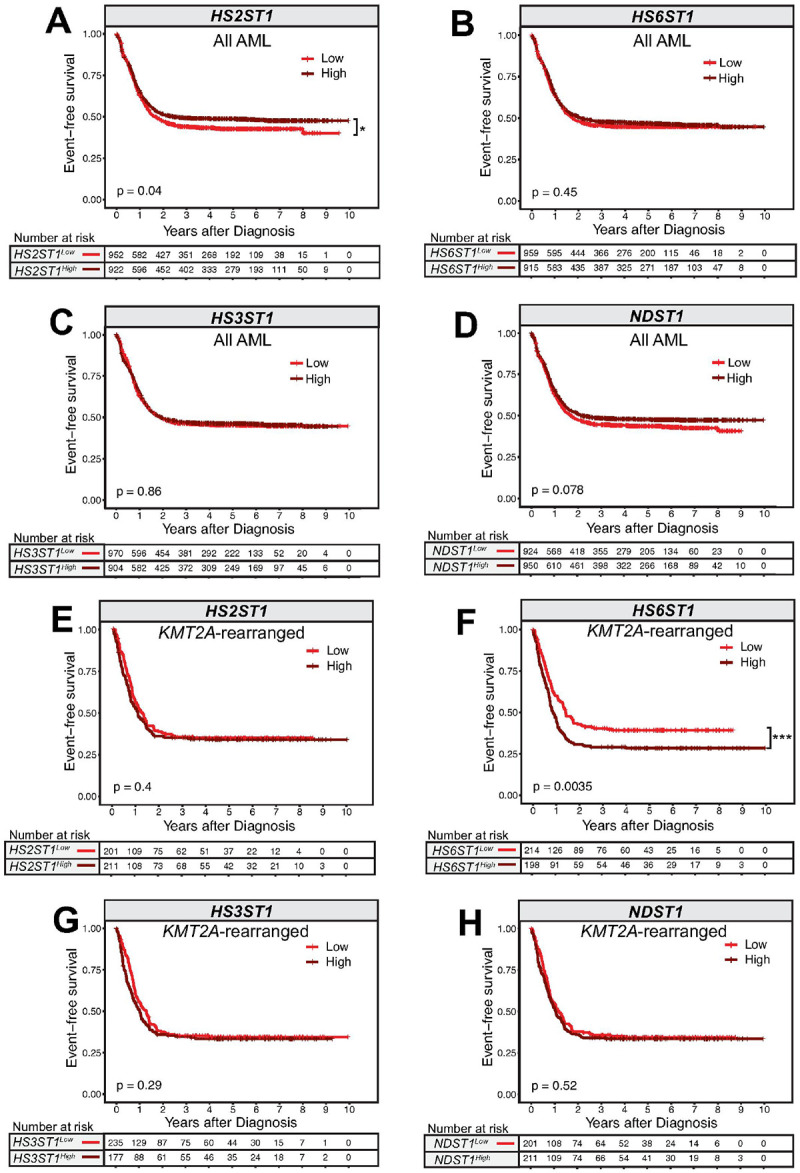
High *HS6ST1* expression predicts poor event-free survival in *KMT2A-*rearranged AML. TARGET patient event free survival data across leukemia subtypes was analyzed based on above-median (high) or below-median (low) expression of **(A)** *HS2ST1*, **(B)** *HS6ST1*, **(C)***HS3ST1*, or **(D)** *NDST1* in bone marrow mononuclear cells from *de novo* patients (*n*=*1,874 AML patients, statistics denote the log-rank test*) TARGET patient event free survival data among patients with *KMT2A*-rearranged AML was analyzed based on above-median (high) or below-median (low) expression of **(E)** *HS2ST1*, **(F)***HS6ST1*, **(G)** *HS3ST1*, or **(H)** *NDST1*.*(n*=*412 KMT2A-rearranged AML patients, statistics denote log-rank tests*).

We next assessed whether heparan sulfotransferase gene expression is associated with differential AML patient outcomes. We classified TARGET AML patients according to their bone marrow expression of *HS2ST1*, *HS6ST1*, *HS3ST1*, and *NDST1* relative to the cohort median. Among all AML patients, individuals with lower *HS2ST1* expression had significantly worse event-free survival than those with high *HS2ST1* expression, but overall survival outcomes were similar (Fig. 2A; **Supplemental Fig. 1A**). Overall survival (**Supplemental Fig. 1B-D**) and event-free survival (Fig. 2B–D) were similar in AML patients regardless of *HS6ST1, HS3ST1*, or *NDST1* expression levels. However, among AML patients with *KMT2A*-rearrangements, increased *HS6ST1* expression correlated with significantly worse event-free and overall survival compared to patients expressing less *HS6ST1* (Fig. 2F; **Supplemental Fig. 1F**). *HS2ST1, HS3ST1*, and *NDST1* expression did not stratify patients harboring *KMT2A*-rearrangements according to differential survival outcomes (Fig. 2E, G–H; **Supplemental Fig. 1E, G-H**). These data indicate that increased *HS6ST1* expression correlates with worse survival outcomes in AML patients with *KMT2A-*rearrangements.

### Depletion of heparan sulfotransferases remodels the AML transcriptome

Previous studies highlight that heparan sulfation controls cancer cell functions (13, 14, 19, 26), leading us to hypothesize that heparan sulfation may regulate AML cells to support disease progression. To test this, we used CRISPR-Cas9 to knockdown *HS2ST1* and *HS6ST1* in the MOLM-13 cell line, which harbors *KMT2A-MLLT3* and *FLT3*-ITD mutations. Compared to sgControl cells, sg*HS2ST1* and sg*HS6ST1* cells had decreased HS2ST1 and HS6ST1 protein expression, respectively, and high indel contribution in target genes. (**Supplemental Fig. 2A-D**).

**Figure 3:**
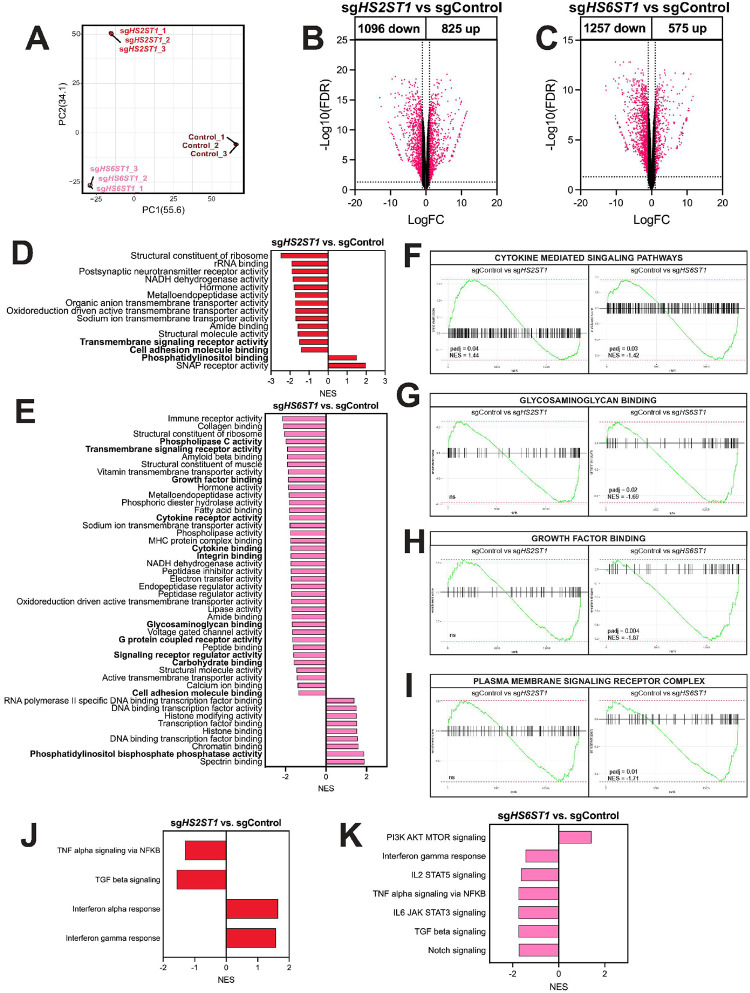
Ablating *HS2ST1* and *HS6ST1* transcriptionally reprograms AML cells. **(A)** Principal component analysis generated from bulk RNA sequencing data from sgControl, sg*HS2ST1,* and sg*HS6ST1* MOLM-13 cells; each point represents one technical replicate. Volcano plot showing differentially expressed genes comparing sgControl to **(B)**sg*HS2ST1* and **(C)** sg*HS6ST1* MOLM-13 cells. Pathways in the molecular function gene set significantly differently expressed in **(D)**sg*HS2ST1* and **(E)** sg*HS6ST1* cells compared to sgControl cells using Gene Set Enrichment Analysis. Bolded terms indicate pathways related to known heparan sulfate functions. Leading edge analysis of **(F)**cytokine mediated signaling pathways, **(G)** glycosaminoglycan binding, **(H)**growth factor binding, and **(I)** plasma membrane signaling receptor complex gene sets. Pathways in the hallmark gene set significantly differently expressed in **(J)** sg*HS2ST1* and **(K)** sg*HS6ST1* MOLM-13 cells compared to sgControl MOLM-13 cells. (*n*=*3 biological replicates; statistics show adjusted p-values*).

Bulk RNA sequencing and principal component analysis revealed sgControl, sg*HS2ST1*, and sg*HS6ST1* cells cluster distinctly, suggesting unique transcriptional profiles (Fig. 3A). Compared to sgControl cells, 1096 genes were downregulated, and 825 genes were upregulated in sg*HS2ST1* cells (Fig. 3B). In sg*HS6ST1* cells, 1257 downregulated genes and 575 upregulated genes were detected compared to sgControl cells (Fig. 3C). Gene Set Enrichment Analysis revealed differentially enriched heparan sulfate proteoglycan-dependent processes in sg*HS2ST1* and sg*HS6ST1* cells compared to sgControl cells (Fig. 3D–E). More specifically, sg*HS2ST1* cells were negatively enriched for gene sets associated with transmembrane signaling receptor activity and cell adhesion molecule binding and positively enriched for phosphatidylinositol binding and cytokine mediated signaling pathways (Fig. 3D, F). sg*HS2ST1* cells were also negatively enriched for TNF-α and TGF-β signaling and positively enriched for interferon alpha and gamma response hallmark gene sets (Fig. 3J). sg*HS6ST1* cells were negatively enriched for gene sets associated with growth factor binding, cytokine receptor activity, cytokine binding, glycosaminoglycan binding, signaling receptor regulator activity, plasma membrane signaling receptor complex, and carbohydrate binding compared to sgControl cells (Fig. 3E, F–I). sg*HS6ST1* cells were also negatively enriched for the interferon gamma response, IL2-STAT5, TNF-α, IL6-JAK-STAT3, TGF-β, and Notch signaling hallmark gene sets and positively enriched for PI3K/AKT/MTOR genes (Fig. 3K). These data indicate that heparan sulfate modifications catalyzed by *HS2ST1* and *HS6ST1* have distinct and farreaching effects on proteoglycan-mediated signaling processes in AML cells.

#### Depletion of HS2ST1 increases AML bone marrow burden in vivo

**Figure 4:**
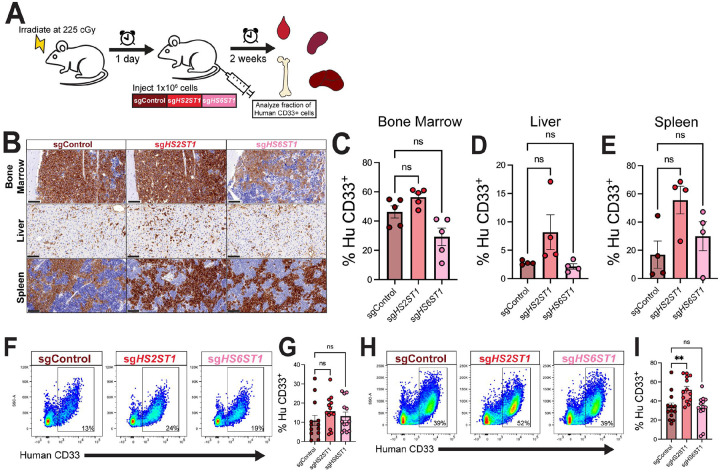
Depletion of *HS2ST1* promotes AML burden *in vivo*. **(A)** Experimental design for *in vivo* AML burden study. **(B)**Micrographs of femurs, livers, and spleens of mice at day +14 after transplant with sgControl, sg*HS2ST1*, or sg*HS6ST1* MOLM-13 cells stained with an anti-human CD33 antibody. Scale bar = 100 μm. Area fraction quantification of human CD33+ leukemic burden in **(C)** femurs, **(D)** livers, and **(E)** spleens (n=*4–5 mice per organ, statistics denote Brown-Forsythe and Welch ANOVA with Dunnet correction; error bars denote SEM*). **(F)**Representative flow cytometry scatter plots and **(G)** quantification of peripheral blood human CD33 burden from xenografts at day +14. (n=13 mice) **(H)**Representative flow cytometry scatter plots and **(I)** quantification of bone marrow human CD33 burden from xenograft at day +14. (n=13 mice; statistics denote one-way ANOVAs with Holm-Sidak corrected t-tests; **p<0.01; error bars denote SEM).

Signaling-related pathways identified from our RNA sequencing analyses are known to impact cancer cell proliferation, leading us to test the potential function of heparan sulfation in AML cell growth (27–29). We tested the impact of *HS2ST1* and *HS6ST1* expression on AML growth *in vivo* using xenograft studies. sgControl, sg*HS2ST1*, or sg*HS6ST1* cells were intravenously transplanted into irradiated NSG mice, and AML burden was quantified using quantitative histology and flow cytometry at 2 weeks-post injection (Fig. 4A). Bone marrow immunohistochemistry showed similar AML burden between all groups, however there was a trend towards increased burden in sg*HS2ST1-*transplanted animals (Fig. 4B–C). Spleen, liver, and peripheral blood AML burden was similar in all groups (Fig. 4B, D–G). Flow cytometry showed a significant increase in AML bone marrow burden upon transplant with sg*HS2ST1* cells compared to sgControl cells (Fig. 4H–I). These data reveal that depletion of *HS2ST1*, but not *HS6ST1*, enhances AML bone marrow burden *in vivo*.

We also assessed cell homing by transplanting sgControl, sg*HS2ST1*, and sg*HS6ST1* into NSG mice (225 cGy) and analyzing AML burden 16 hours post-transplant (**Supplemental Fig. 3A**). sgControl, sg*HS2ST1*, or sg*HS6ST1* cell homing to the bone marrow or spleen were similar (**Supplemental Fig. 3B-C**), suggesting that *HS2ST1*-mediated changes in AML bone marrow burden occur independently of homing.

#### Higher HS6ST1 expression correlates with increased relapse risk in KMT2A-r AML patients

**Figure 5:**
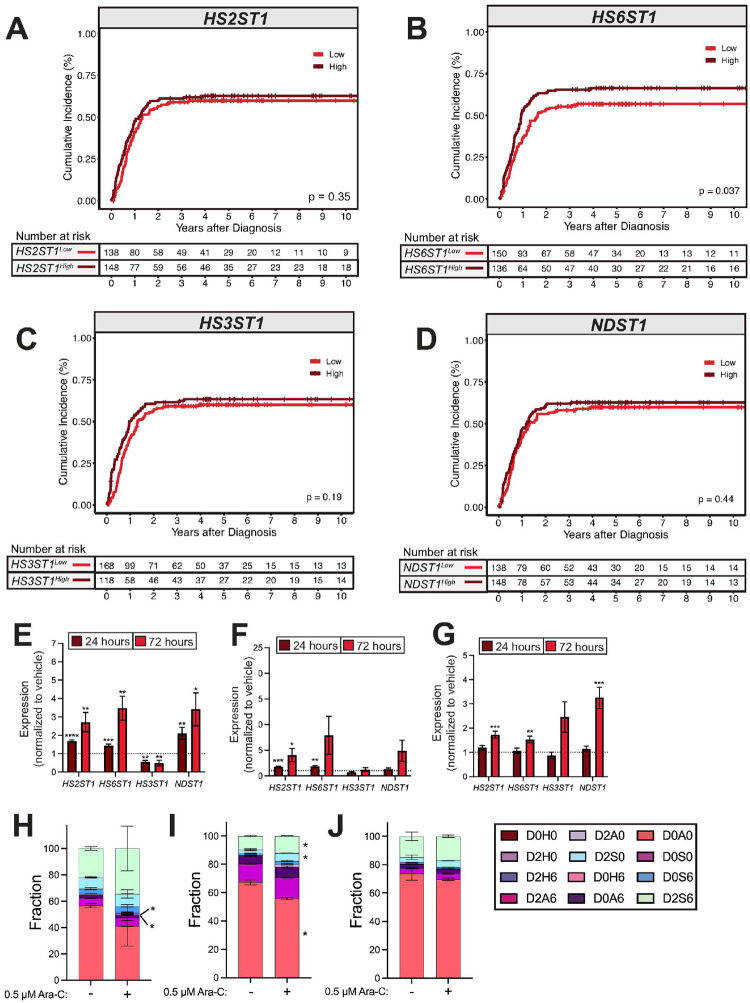
Cytarabine treatment remodels heparan sulfotransferases gene expression in AML. TARGET AML patient relapse risk for patients with *KMT2A-*rearranged AML based on above-median (high) or below-median (low) expression of **(A)** *HS2ST1,* **(B)***HS6ST1,* **(C)** *HS3ST1,* or **(D)** *NDST1 (n*=*286 KMT2A-rearranged AML patients)*. Expression of *HS2ST1, HS6ST1, HS3ST1,* and *NDST1* after a 24-hour treatment with 0.5 μM Ara-C in **(E)** MOLM-13, **(F)** Kasumi-1, or **(G)** THP-1 cells. Gene expression was normalized to the vehicle-treated sample (dotted line). (*n*=3–9 biological replicates, Statistics denote an unpaired t-test comparing Ara-C treated samples to vehicle treatment; *p<0.05, **p<0.01, ***p<0.001, ****p<0.0001; error bars denote SEM). Liquid chromatography mass spectrometry analysis for specific heparan sulfate disaccharides expressed by **(H)** MOLM-13, **(I)**Kasumi-1, and **(J)** THP-1 cells after a 72-hour treatment with DMSO or 0.5 μM Ara-C. (*n*=2–3 biological replicates per condition per cell line; statistics denote nested ANOVAs; *p<0.05; error bars denote SEM).

Disease relapse driven by chemotherapy-resistant AML cells is a major barrier to long term patient survival (30). We therefore interrogated whether heparan sulfotransferase expression correlated with relapse risk in AML. Low *HS2ST1* expression predicted higher relapse risk across AML subtypes (**Supplemental Fig. 4A**). There were no significant differences in relapse risk based on *HS3ST1, HS6ST1*, and *NDST1* expression across AML subtypes (**Supplemental Fig. 4B-D**). *HS2ST1*, *HS3ST1* and *NDST1* expression were not correlated with *KMT2A-*rearranged patient relapse risk (Figs. 5A, C–D). In contrast, high bone marrow *HS6ST1* expression was associated with significantly increased relapse risk in *KMT2A-*rearranged AML patients (Fig. 5B). These data demonstrate that high *HS6ST1* correlates with increased relapse in patients with *KMT2A*-rearranged leukemias.

### Cytarabine treatment alters heparan sulfotransferase gene expression in AML cell lines

Previous studies show that the heparan sulfate landscape differs in therapy-refractory residual breast cancer tumor cells compared to the primary tumor (20). These findings led us to test whether therapy-refractory AML cells express distinct heparan sulfate landscapes that enable them to resist chemotherapy and promote relapse. MOLM-13 cells expressed increased *HS2ST1, HS6ST1*, and *NDST1* and decreased *HS3ST1* at 24- and 72-hours post-Ara-C treatment compared to vehicle-treated controls (Fig. 5E). Kasumi-1 cells had significantly increased *HS2ST1* and *HS6ST1* expression at 24-hours post-Ara-C treatment compared to vehicle, but *HS3ST1* and *NDST1* expression were unchanged (Fig. 5F). *HS2ST1, HS6ST1*, and *NDST1* expression were significantly elevated in THP-1 cells at 72 hours post-Ara-C treatment compared to vehicle controls (Fig. 5G). These data suggest that chemotherapy resistant AML cells express different heparan sulfate transcriptomic profiles compared to their chemosensitive counterparts.

Liquid chromatography mass spectrometry revealed significantly increased D2H6 and decreased D2A0 disaccharides in MOLM-13 cells treated with Ara-C compared to vehicle-treated cells (Fig. 5H). In Kasumi-1 cells, there was significantly decreased D0A0 and significantly increased D2S0 and D2S6 disaccharides upon Ara-C treatment (Fig. 5I). There were no significant changes in the heparan sulfate disaccharide composition in THP-1 cells after treatment with Ara-C (Fig. 5J). These data show that Ara-C remodels the AML heparan sulfate landscape at the glycan level, suggesting that the precise structure of heparan sulfate may influence AML cells chemotherapy responses.

#### HS6ST1 depletion promotes sensitivity to cytarabine

**Figure 6:**
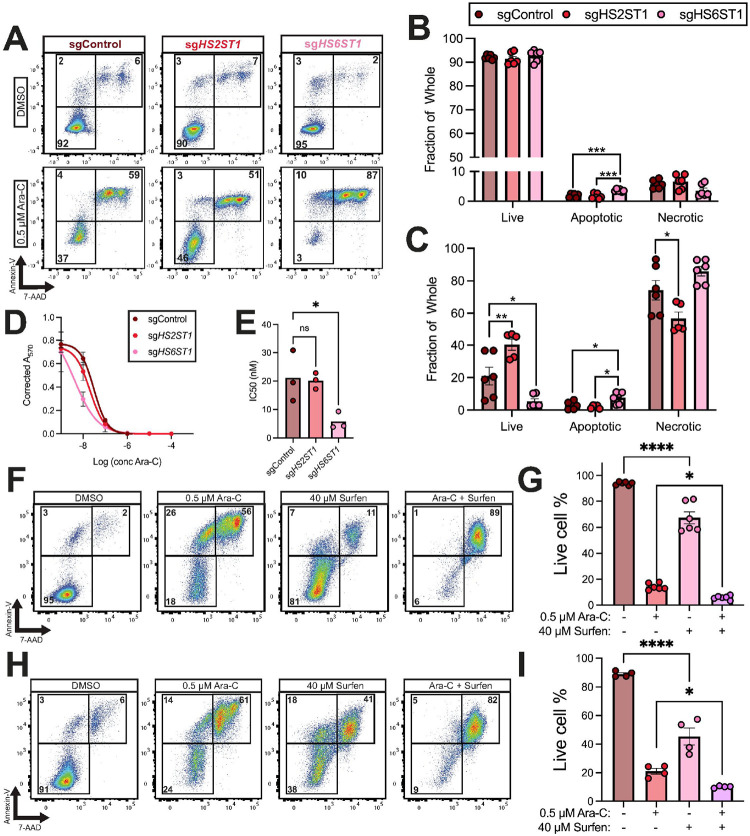
*HS6ST1* promotes cytarabine resistance in MOLM-13 cells. **(A)** Representative flow cytometry scatter plots depicting Annexin-V/7-AAD staining of sgControl, sg*HS2ST1,* and sg*HS6ST1* MOLM-13 cells treated with DMSO or 0.5 μM Ara-C for 72 hours and quantification of live, apoptotic, and necrotic cells in **(B)**vehicle-treated MOLM-13 cells or **(C)** Ara-C-treated MOLM-13 cells (*n*=*6 biological replicates across n*=*3 independent experiments*). **(D)** Ara-C IC50 curves and **(E)** quantification from sgControl, sg*HS2ST1,* and sg*HS6ST1* MOLM-13 cells (*n*=3 independent experiments, statistics denote a one-way ANOVA with Holm-Šidák correction; *p<0.05). **(F)**Representative flow cytometry scatter plots from MOLM-13 cells treated with DMSO, 0.5 μM Ara-C, 40 μM surfen, or both for 72-hours and analyzed using Annexin-V/7-AAD staining, and **(G)** quantification (n=6 biological replicates across 3 independent experiments). **(H)** Representative flow cytometry scatter plots from THP-1 cells treated with DMSO, 0.5 μM Ara-C, 40 μM surfen, or both for 72-hours and analyzed using Annexin-V/7-AAD staining, and **(I)**quantification (*n*=6 biological replicates across 3 independent experiments; statistics denote a one-way ANOVA with Holm-Šidák correction; *p<0.05, ****p<0.0001; error bars denote SEM).

Because Ara-C treatment increases heparan sulfotransferase gene expression, we investigated whether *HS2ST1* or *HS6ST1* regulate AML sensitivity to Ara-C. After 72 hours, Vehicle-treated sgControl, sg*HS2ST1*, and sg*HS6ST1* cells exhibited similar levels of live and necrotic cells, while sg*HS6ST1* cells had slightly increased apoptotic cells (Fig. 6A–B). However, upon Ara-C treatment, sg*HS2ST1* cells had significantly more live cells than sgControl cells, suggesting they are more resistant to chemotherapy (Fig. 6C). sg*HS6ST1* cells had significantly fewer live cells compared to sgControl cells, accompanied by increased apoptotic cells, indicating they are more chemosensitive (Fig. 6C). Consistent with these data, there was a four-fold reduction in the Ara-C IC50 for sg*HS6ST1* cells compared to sgControl cells (Fig. 6D–E). Together, these data show that *HS6ST1* and *HS2ST1* distinctly regulate AML chemotherapy sensitivity.

Surfen is a small molecule antagonist of heparan sulfate (31). To evaluate whether heparan sulfate blockade is therapeutically effective for AML, we treated MOLM-13 and THP-1 cells with surfen alone and in combination with Ara-C. Compared to vehicle treatment, surfen treatment decreased cell viability in MOLM-13 and THP-1 cells, but not to the same level as Ara-C. However, combinatorial treatment of MOLM-13 or THP-1 cells with Ara-C and surfen significantly decreased cell viability compared to Ara-C treatment alone (Fig. 6F–I). These data show that heparan sulfate antagonism synergizes with Ara-C to promote AML cytotoxicity.

#### HS6ST1 is promotes TGF-β1 signaling to regulate AML survival upon chemotherapy treatment

**Figure 7:**
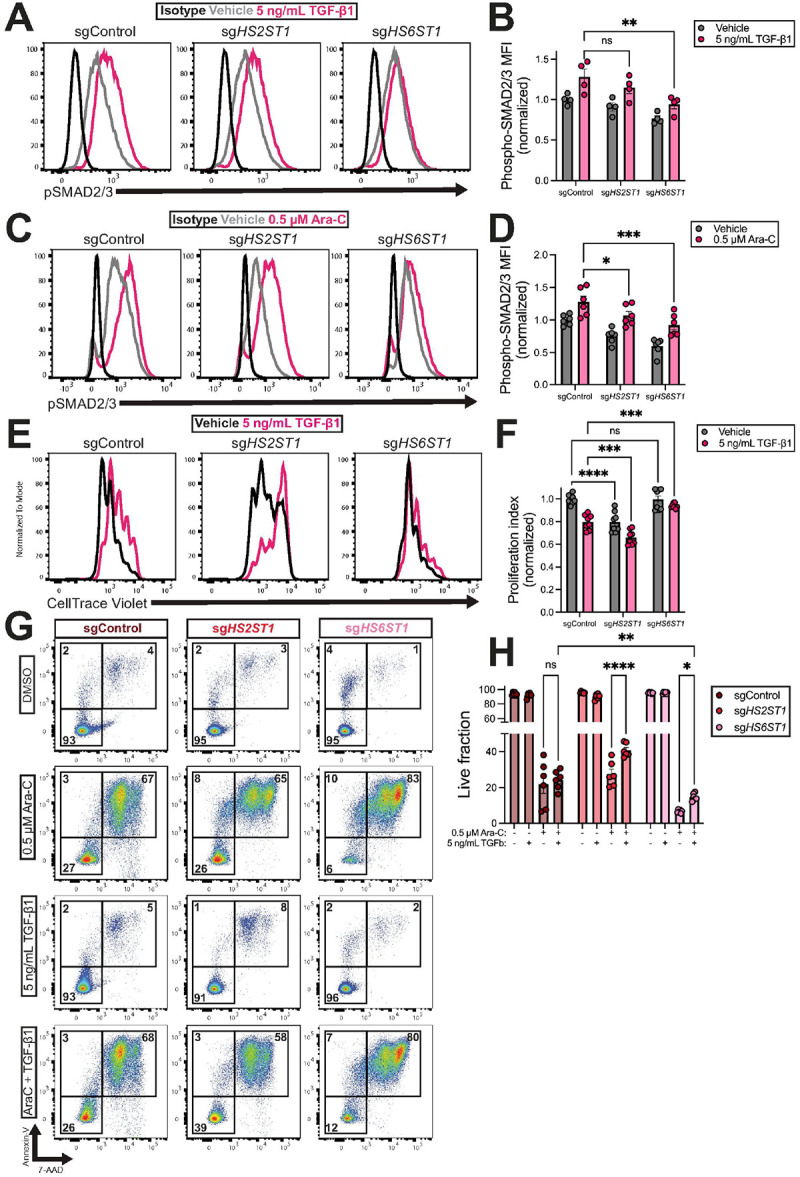
*HS6ST1* promotes TGF-β1 signaling and chemotherapy resistance in AML. **(A)** Representative flow cytometry histograms depicting phospho-SMAD2/3 expression in sgControl, sg*HS2ST1*, and sg*HS6ST1* MOLM-13 cells treated with 5 ng/mL TGF-β1 or vehicle for 2 hours and **(B)** quantification. **(C)** Representative flow cytometry histograms depicting phospho-SMAD2/3 expression in sgControl, sg*HS2ST1*, and sg*HS6ST1* MOLM-13 cells treated with 0.5 μM Ara-C or vehicle for 24 hours and **(D)** quantification (*n*=4–6 biological replicates across 2–3 independent experiments, statistics denote a two-way ANOVA with Holm-Šidák corrected t-tests; *p<0.05, ***p<0.001). **(E)** Representative flow cytometry histograms depicting CellTrace Violet dye levels in sgControl, sg*HS2ST1,* and sg*HS6ST1* MOLM-13 cells stained with CellTrace Violet and grown in complete media or complete media + 5 ng/mL TGF-β1 for 72 hours. **(F)**Proliferation index was calculated from CellTrace Violet data using FlowJo V10.10 (*n*=6 biological replicates across 3 independent experiments; Statistics denote a two-way ANOVA with Holm-Šidák t-tests; **p<0.01, ****p<0.0001). **(G)** Representative flow cytometry scatter plots for sgControl, sg*HS2ST1,* and sg*HS6ST1* MOLM-13 cells treated with DMSO, 0.5 μM Ara-C, 5 ng/mL TGF-β1, or both Ara-C and TGF-β1 for 72-hours and stained using Annexin-V/7-AAD and (**H)** analysis of live, apoptotic, and necrotic cells (*n*=6 biological replicates across 3 independent experiments; statistics denote a two-way ANOVA with Holm-Šidák correction; *p<0.05, ****p<0.0001; error bars denote SEM).

Our RNA sequencing data indicates that compared to sgControl cells, sg*HS6ST1* cells were negatively enriched for pathways related to growth factor signaling including TGF-β1 (Fig. 3C). Bone marrow TGF-β1 levels are significantly increased in relapsed/refractory AML patients, and high *TGFB1* expression predicts adverse prognoses (32, 33). TGF-β1 supplementation promotes AML chemoresistance *in vitro* by inducing a quiescent-like G_0_ shift (34, 35). We therefore hypothesized that depleting *HS6ST1* may impact TGF-β1 signaling in AML, rendering cells more susceptible to Ara-C. Using intracellular flow cytometry, we measured phospho-SMAD2/3, a response element downstream of TGF-β1. TGF-β1 stimulation increased phospho-SMAD2/3 levels in sgControl and sg*HS2ST1* compared to vehicle treatment (Fig. 7A–B). However, phospho-SMAD2/3 expression upon TGF-β1 stimulation of sg*HS6ST1* cells was muted compared to sgControl cells (Fig. 7A–B). Similarly, Ara-C treatment induced SMAD2/3 phosphorylation in all cell lines relative to vehicle treatment (Fig. 7C–D). However, phospho-SMAD2/3 expression was significantly lower in Ara-C-treated sg*HS2ST1* and sg*HS6ST1* than Ara-C-treated sgControl cells (Fig. 7C–D). We next measured cell divisions at baseline and upon TGF-β1 stimulation using CellTrace Violet staining. sg*HS2ST1* cells had a lower proliferation index than sgControl cells at baseline and after stimulation with TGF-β1. sg*HS6ST1* proliferation index was not significantly different from sgControl cells at baseline, but it was significantly higher than sgControl cells after TGF-β1 treatment (Fig. 7E–F). These data indicate that depletion of *HS6ST1* decreases TGF-β1-mediated SMAD2/3 activation and supports cell proliferation.

As TGF-β1 is known to promote AML chemotherapy resistance (32), we reasoned that sg*HS6ST1* cells may exhibit reduced cell survival abilities compared to sgControl cells due to an impaired ability to respond to TGF-β1. To test this, cells were treated with Ara-C and TGF-β1 and cell death was quantified with Annexin-V/7-AAD staining. TGF-β1 treatment alone had no impact on cell survival (Fig. 7G–H). However, TGF-β1 treatment in combination with Ara-C promoted the viability of sg*HS2ST1* cells and sg*HS6ST1* cells compared to Ara-C treatment alone. However, even with this increase in viability, sg*HS6ST1* cells do not achieve the level of cell survival detected in sgControl cells. These data suggest that *HS6ST1* cells have an impaired ability to respond to TGF-β1 signaling, which sensitizes cells to Ara-C. Taken together, our findings indicate that *HS6ST1* regulates AML chemotherapy resistance, and depletion of *HS6ST1* can sensitize AML cells to Ara-C, while impairing their ability to respond to TGF-β1 stimuli.

## DISCUSSION

We reported previously that the heparan sulfate proteoglycan syndecan-2 regulates normal hematopoietic stem cell functions (36); however, the effect of heparan sulfates in malignant hematopoiesis had not been fully analyzed. Our study identified a crucial link between the heparan sulfate biosynthesis machinery and AML patient survival outcomes and chemotherapy resistance. This work adds to a growing body of literature defining the roles of heparan sulfation in cancer physiology, such as work from others showing that 6-*O* sulfation is necessary for survival-promoting signaling in treatment refractory dormant breast cancer residual tumor cells (20). Further, the presence and structures of syndecan-1 heparan sulfate chains regulate multiple myeloma proliferation and signaling (37, 38). Our findings provide a foundation to better understand the function of heparan sulfates in other hematological malignancies and hematopoietic disorders.

Mechanistically, we show that *HS6ST1* regulates TGF-β1 signaling to support AML cell survival upon Ara-C. Several studies highlight critical functions for heparan sulfate proteoglycans in regulating TGF-β1 signaling (39–42). Work on hepatocellular carcinoma demonstrated a connection between the 6-*O* heparan sulfate endosulfatase *SULF1* and TGF-β1 signaling (41). However, HS6ST1 decorates heparan sulfates during the biosynthesis process in the endoplasmic reticulum, while SULF1 acts extracellularly after heparan sulfate proteoglycan trafficking. Therefore, the push and pull of heparan sulfate biosynthesis and post-synthesis processing should be carefully analyzed in the future to resolve the distinct contributions of these processes in AML and how they change during disease.

Correlations between TGF-β1 and AML survival outcomes are well-characterized, but inhibiting TGF-β1 receptors in AML can induce expression of drug efflux pumps, weakening treatment efficacy (32, 33, 35, 43). Targeting heparan sulfate to extrinsically reprogram TGF-β1 signaling in AML cells could represent a therapeutic avenue to improve patient outcomes. In our study, we show that the heparan sulfate antagonist surfen promotes AML cell killing alone and in combination with Ara-C. Other groups showed that surfen decreases tumorigenicity of Ewing sarcoma cells by reprogramming growth factor signaling (44). Surfen can also inhibit glioblastoma invasion by blocking chondroitin sulfate, another type of glycosaminoglycan (45). Further, others have shown heparan sulfate mimetics can therapeutically target colorectal cancer stem cells (46). Our results build on these studies by supporting the notion that targeting extracellular glycans represents a viable way to inhibit blood cancer progression, starting with therapeutic relevance in AML.

We showed that AML patient peripheral blood mononuclear cells express heparan sulfate disaccharide profiles that are distinct from normal donor cells. However, open questions regarding heparan sulfate structural changes in AML remain. A landmark study using single-chain variable fragment antibodies targeting differentially modified heparan sulfate showed that distinct heparan sulfate patterns can identify hematopoietic cells primed for different lineages (47). However, heparan sulfate chains are between 40 and 300 sugar residues long, and sulfate modifications and patterning are both important to coordinate growth factor signaling (12, 17). While disaccharide analysis can provide insight into which modifications are present, tools have yet to be created to depict heparan sulfate patterning and chain length with sufficient resolution to accurately discern complete structural motifs. The field will benefit from more robust mass spectrometry methods to characterize the structures of entire heparan sulfate chains, another layer of information that likely informs AML cell responses to chemotherapy.

## Supplementary Files

This is a list of supplementary files associated with this preprint. Click to download.

- SupplementalMaterialCombined.pdf
